# War Babies

**Published:** 1915-09

**Authors:** Bessie Drsydale


This little pamphlet from Mrs. Bessie Drysdale, who is one of the honorable secretaries of the Malthusian League, of England, came to our desk a few days ago. It is an able argument for "Quality and not quantity" in the human race. Write Mrs. Drysdale for a sample copy of "The Mathusian"; you need it. The address is Queen Anne's Chambers, Westminster, S. W., London, England.—ED.

### WAR BABIES.

The writer of this leaflet believes that the greatest enemy to the health, independence and well-being of the working classes is their ignorance of the knowledge that families can and should be limited by lawful and healthful methods to the number that parents can do justice to.

No other consideration ought to weigh with them; no matter whether they are urged by church or State to bring large numbers into the world, they should insist on the right to have only such children as their wages or health will enable them to raise in decent conditions and with a decent outlook for the future.

During this time of war especially, parents should consider more carefully than ever before whether they are in a position to guarantee the well-being of any fresh additions to their family. Wages have risen, it is true, but no so fast as the price of food and necessaries of every kind. Many men are making double their usual wages on "war work," but do they realize that after the war they may have a difficulty in getting even their usual wages when the war work ceases and the men return from the front to flood the labor market? This war will have to be paid for, and working people will suffer as well as all other classes in the depression which will follow the false "prosperity" of the present time when we are using up our national savings.

*This is the time when everybody should lay by for the rainy day* which will surely come, and the most profitless expenditure of all others is to call an innocent being into life, who is likely to be born to suffer or die in its early childhood from want of the care and comforts necessary to its existence. Since this terrible war commenced, people have been saying that, in view of the huge losses of our men at the front, large numbers of children should be born to replace them. Such careless reasoning as this needs to be warned against. Here are a few figures which will disprove such arguments.

1. We are not likely to lose more than 60,000 British men by *death* in the first year of this war. (It will be remembered that casualties included wounded and missing as well as deaths.)

2. Against this we have 1,100,000 babies born in an ordinary year in the United Kingdom, so that there are twenty children to replace each man lost.

3. Of this 1,100,000 babies about 120,000 die within the first year of life, even in ordinary times. They are mostly among the children of the poorer classes, which points to the want of the proper conditions to keep them alive.

4. Now, if only half of this 120,000 were kept alive, as they certainly could be if families were small, there are enough of them to replace all the soldiers who are killed, without counting nearly a million babies who outlived their first year.

*So you see, there is little need to cry aloud for more and still more babies while not only this huge number are born, but while so many of those born are wasted by dying before they are a year old.*

Again, the men who are fighting for us would have been working for the necessaries of life, had there been no war. So that there are less people now to get necessities, such as food, and for this reason, as well as the difficulties of transport, prices are rising. Babies, on the other hand, consume necessaries without being able to produce them, and at such a time we have no right to add to our difficulties in this way.

*Not only, however, do unwanted babies tend to die, but, while they live, they make it more difficult for the older children to be cared for properly—many of them are sure to be ill or die, who*

*could have been saved if the mothers had been able to give them more and better food and attention.*

To those young married women who are yet without children one might suggest that in these times of stress, both for their own and the national need, they should take the men's places as producers, and wait till the end of the war or till the future is brighter before having their children.

Nowadays more and more is being done to take the responsibilities of rearing their children out of the hands of the poorer parents. State education, compulsory medical inspection, maternity endowments, and dozens of voluntary associations for mother and child welfare, whose avowed object is the preservation of life and health among the poorer people, are constanly thrusting themselves into the homes and lives of working people for the object of encouraging and keeping alive huge numbers in that class. The families of all above the working classes are small, and the parents would refuse and resent any interference in their private affairs, or any offers of charity. *It is only fair to warn working men and women that if they listen to those who would encourage them to have families beyond the number they can raise independently, not only will they thus give up their splendid birthright of independence, but they will lay up a future of misery for their children, who, by overcrowding the labor market will always be slaves of their masters, whether those masters are the private owners of capital of our present system, or the officials of a Socialistic State controlling production and distribution. Labor, to be valuable and free, must, to a certain extent, be limited. What is evidently wanted to make things comfortable for everybody is to have less mouths and more to put in them; or, in other words, workers should limit their numbers and not their output.*

BESSIE DRYSDALE.

---

We desire to call attention to the advertisement of the Griffith Drug Company which appears in this issue of the JOURNAL. The invitation to make the store your headquarters while in Austin is a genuine one, and you cannot find a more delightful place—the store is one of the largest and most up-to-date in the city, the proprietors and the clerks are courteous and obliging and you will always find some members of the profession there. Try it next time you come to town.

Dr. Joe Gilbert, of Austin, has recently installed a complete up-to-date X-ray laboratory with the Scheidel's Western Ten K. V. A. transformer and Roentgenoscope complete.

He is also prepared to do cystoscopic work and clinical pathological work. Dr. G. H. Gilbert and Dr. John H. Thorne are his associates.

This machine is a beauty, and we feel sure that the doctors in this part of the country will be glad to know where they can have such work done near home.

By the way, who was it said that Austin was a hundred years behind?

